# Supplementary material for: Grain engineering for efficient near-infrared perovskite light-emitting diodes
Source: Nat Commun. 2024 Dec 30;15:10760. doi: 10.1038/s41467-024-55075-3 (PMC11685452; doi:10.1038/s41467-024-55075-3)
Supplement: Supplementary file 1 — Supplementary Information [file 41467_2024_55075_MOESM1_ESM.docx]

Supplementary Information

**Grain engineering for efficient near-infrared perovskite light-emitting diodes**

Sung-Doo Baek^1,16^, Wenhao Shao^1,16^, Weijie Feng^2^, Yuanhao Tang^1^, Yoon Ho Lee^1^, James Loy^3^, William B. Gunnarsson^4^, Hanjun Yang^1,5^, Yuchen Zhang^6^, M. Bilal Faheem^6^, Poojan Indrajeet Kaswekar^6^, Harindi R. Atapattu^7^, Jiajun Qin^8^, Aidan H. Coffey^9^, Jee Yung Park^1,10,11^, Seok Joo Yang^1^, Yu-Ting Yang^1^, Chenhui Zhu^9^, Kang Wang^1,12^, Kenneth R. Graham^7^, Feng Gao^8^, Quinn Qiao^6^, L. Jay Guo^2,13^, Barry P. Rand^4,14^, Letian Dou^1,5,15^*

^1^Davidson School of Chemical Engineering, Purdue University; West Lafayette, IN, USA.

^2^Macromolecular Science and Engineering, University of Michigan, Ann Arbor, MI, USA.

^3^Department of Physics, Princeton University, Princeton, NJ, USA.

^4^Department of Electrical and Computer Engineering, Princeton University, Princeton, NJ, USA.

^5^Department of Chemistry, Purdue University; West Lafayette, IN, USA.

^6^Department of Mechanical and Aerospace Engineering, Syracuse University, Syracuse, NY, USA.

^7^Department of Chemistry, University of Kentucky, Lexington, KY, USA.

^8^Department of Physics, Chemistry and Biology (IFM), Linköping University, Linköping, Sweden.

^9^Advanced Light Source, Lawrence Berkeley National Laboratory, Berkeley, CA, USA.

^10^Department of Chemical and Environmental Engineering, Yale University, New Haven, CT, USA.

^11^Energy Sciences Institute, Yale University, West Haven, CT, USA.

^12^Key Laboratory of Photochemistry, Institute of Chemistry, Chinese Academy of Sciences, Beijing, China.

^13^Department of Electrical Engineering and Computer Science, University of Michigan, Ann Arbor, MI, USA.

^14^Andlinger Center for Energy and the Environment, Princeton University, Princeton, NJ, USA.

^15^Birck Nanotechnology Center, Purdue University; West Lafayette, IN, USA.

^16^These authors contributed equally to this work.

*Corresponding author Email: dou10@purdue.edu.

Table of Contents

[Supplementary Fig. 1. AFM topography of perovskite films 3](#Fig1)

[Supplementary Fig. 2. Perovskite film and PeLED characteristics 4](#Fig2)

[Supplementary Fig. 3.TEM analysis (solvent-engineered device) 5](#Fig3)

[Supplementary Fig. 4. Discrete island – convex dome morphology under AFM 6](#Fig4)

[Supplementary Fig. 5. TeFBTT characteristics 7](#Fig5)

[Supplementary Fig. 6. GIWAXS analysis 8](#Fig6)

[Supplementary Fig. 7. TEM analysis (TeFBTT-treated device) 9](#Fig7)

[Supplementary Fig. 8. Device characteristics of PeLEDs with TeFBTT 10](#Fig8)

[Supplementary Fig. 9. Characteristics of TeFBTT-treated perovskite films 11](#Fig9)

[Supplementary Fig. 10. Perovskite 2D/3D phase ratio estimation 12](#Fig10)

[Supplementary Fig. 11. PeLEDs with other organic cations 13](#Fig11)

[Supplementary Fig. 12. Other representative PeLEDs with TeFBTT 14](#Fig12)

[Supplementary Fig. 13. Reported max EQE and radiance of NIR PeLEDs 15](#Fig13)

[Supplementary Fig. 14. Histograms of nanoscale charge-carrier recombination 16](#Fig14)

[Supplementary Fig. 15. Defect passivation model 17](#Fig15)

[Supplementary Fig. 16. Cross-validation of PeLEDs 18](#Fig16)

[Supplementary Fig. 17. Device characteristics of the cross-validated PeLED 19](#Fig17)

[Supplementary Table 1. Performance summary of reported NIR PeLEDs 20](#Table1)

[Supplementary Note 1. Solution chemistry between 5AVA and FAI 21](#Note3)

[Supplementary Fig. 18. Plausible chemistry involving 5AVA 21](#Fig24)

[Supplementary Fig. 19. GC-MS spectra 22](#Fig25)

[Supplementary Fig. 20. NMR spectra of 5AVA in DMSO 23](#Fig26)

[Supplementary Fig. 21. Reaction between FAI and 5AVA in DMSO 24](#Fig27)

[Supplementary Information for organic synthesis 25](#SI)

[Supplementary Fig. 22. Synthetic scheme for TeFBTT 25](#Fig28)

[NMR spectra 26](#NMR)

[Supplementary Table 2. Crystal data and structure refinement for (TeFBTT)_2_PbI_4_ 31](#Table4)

[Supplementary Note 2. Grain analysis from SEM and AFM 33](#Note1)

[Supplementary Fig. 23. The average height (*H*) determination 33](#Fig18)

[Supplementary Fig. 24. The convex height (*h*_s_) determination 33](#Fig19)

[Supplementary Fig. 25. Particle analysis process 34](#Fig20)

[Supplementary Note 3. Power density calculation for PLQY measurements 35](#Note4)

[Supplementary Fig. 26. Laser power density details 35](#Note4)

[Supplementary Note 4. Optical simulations 36](#Note2)

[Supplementary Fig. 27. Refractive index spectra used for optical simulations 36](#Fig21)

[Supplementary Fig. 28. Simulation models 37](#Fig22)

[Supplementary Table 3. Simulation parameters and results from the periodic model 37](#Table2)

[Supplementary Fig. 29. Specific SEM sections used for the randomly distributed model 38](#Fig23)

[Supplementary Table 4. Simulation results from the randomly distributed model 38](#Table3)

[Supplementary References 39](#Ref)


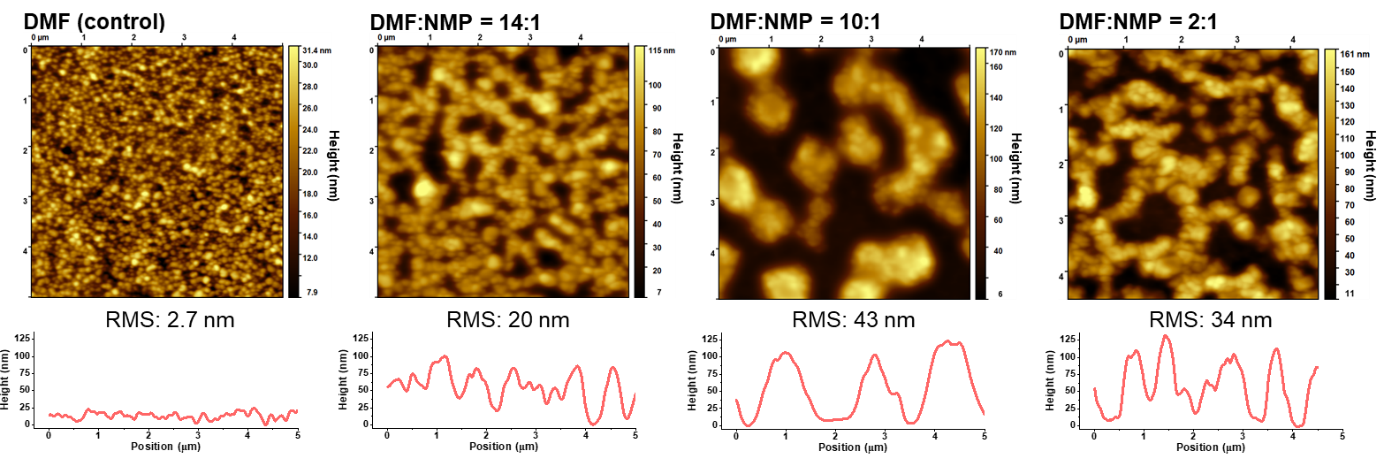


Supplementary Fig. 1. AFM topography of perovskite films with different solvent mixing ratios. Line profiles are included under each image.

**
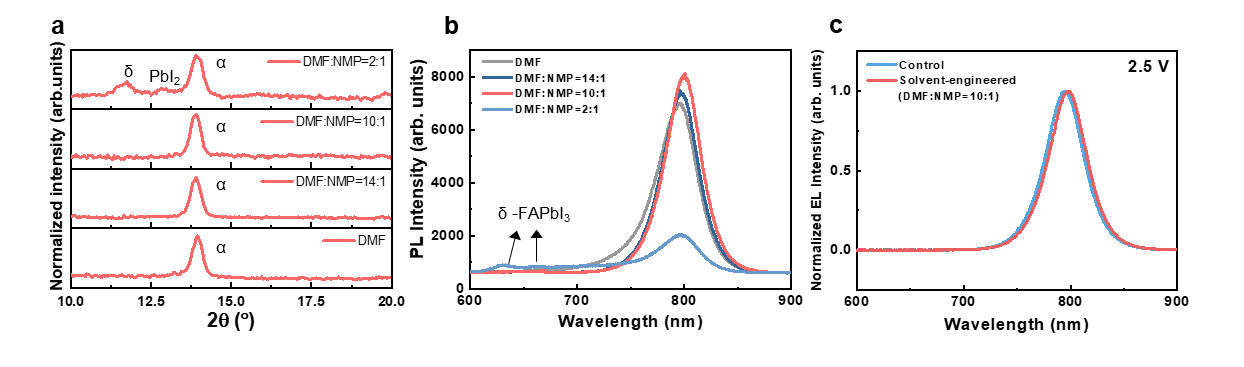
**

**Supplementary Fig. 2. Perovskite film and PeLED characteristics** (a) XRD spectra and (b) PL spectra of perovskite films with varying solvent mixing ratios. (c) EL spectra of PeLEDs with control and solvent-engineered (DMF:NMP = 10:1) films.

**
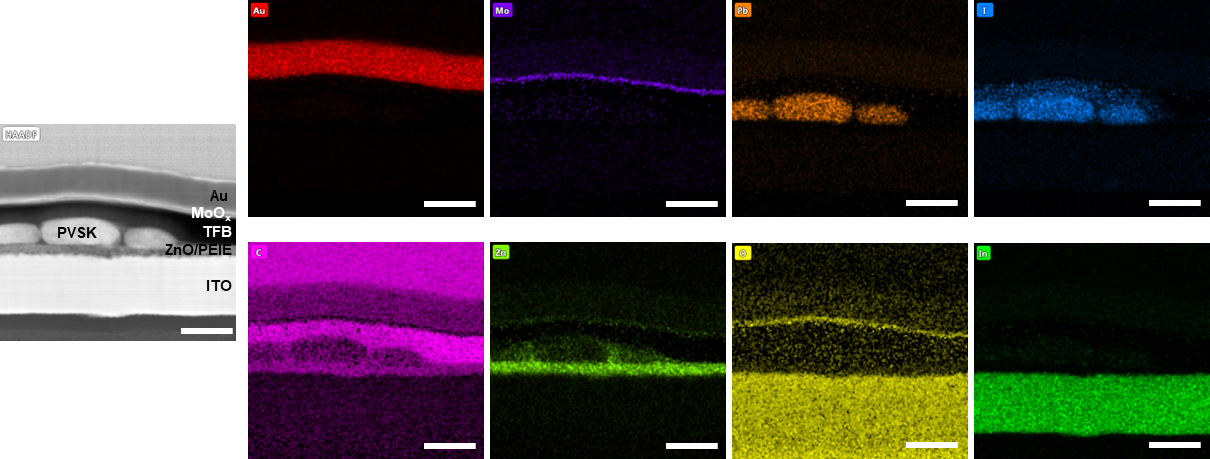
**

**Supplementary Fig. 3. TEM analysis (solvent-engineered device).** Cross-sectional TEM and EDS mapping images of the solvent-engineered device (scale bar: 100 nm).


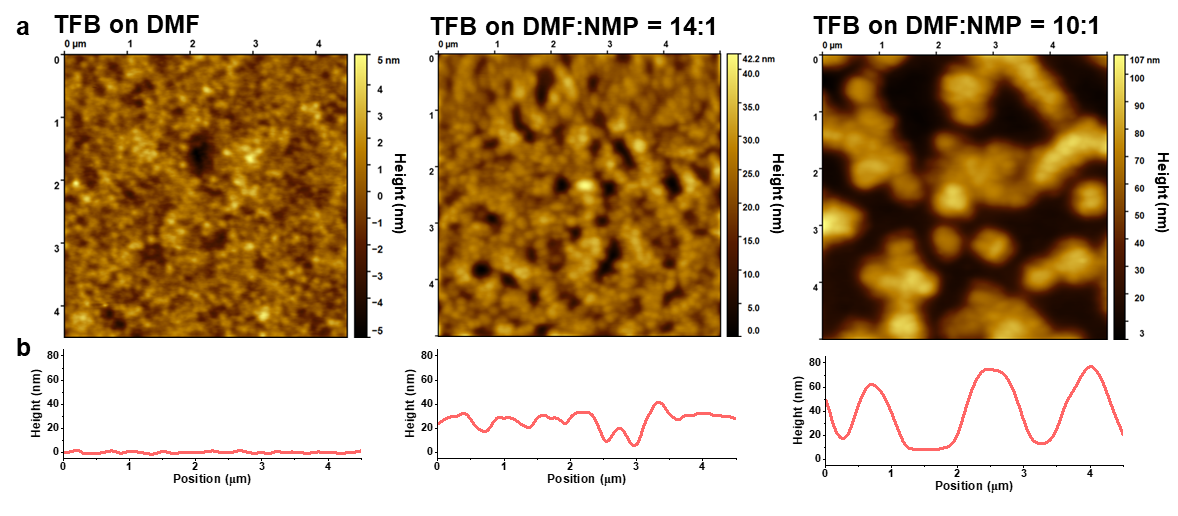


Supplementary Fig. 4. Discrete island – convex dome morphology under AFM. (a) AFM images and (b) height line profiles of TFB on perovskite films under varying solvent mixing ratios.

**
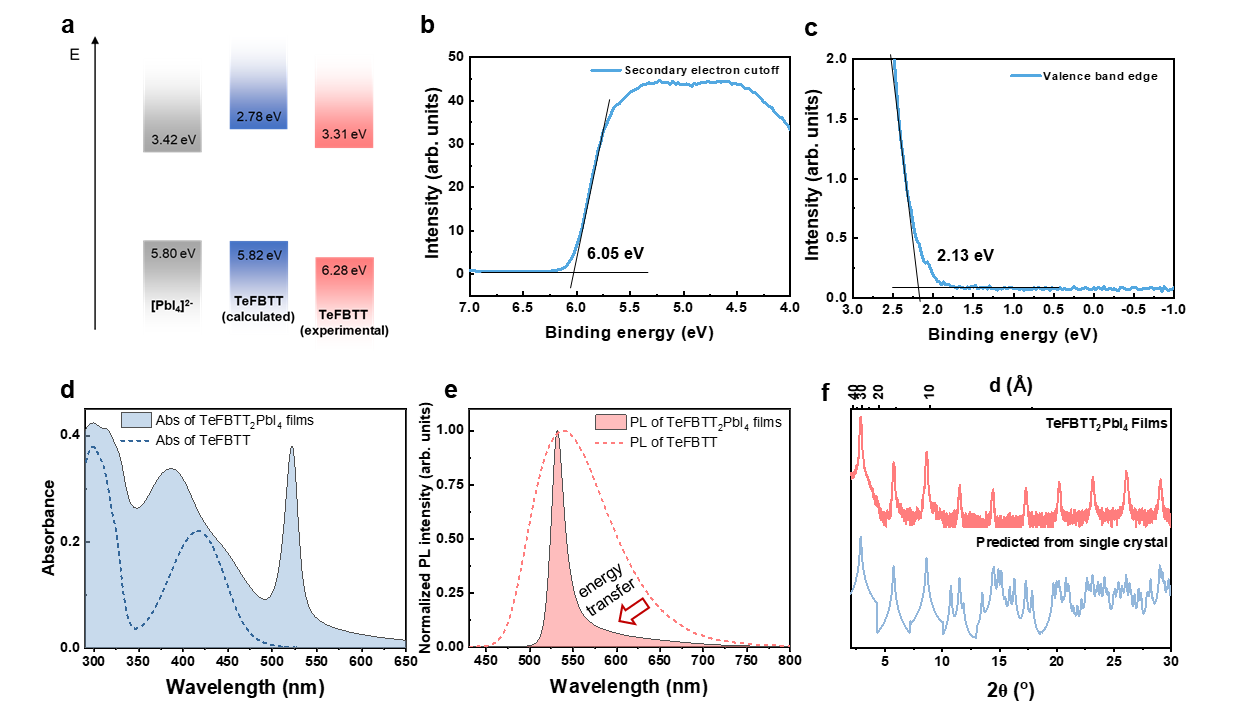
**

**Supplementary Fig. 5. TeFBTT characteristics.** (a) Energy band diagram of [PbI_4_]^2-^ and TeFBTT with calculated and experimental values. [PbI_4_]^2-^ values were sourced from a previous study^1^. (b) Secondary electron cutoff and (c) valence band edge spectra of TeFBTT. (d) Absorption spectra and (e) PL spectra of (TeFBTT)_2_PbI_4_ and TeFBTT. (f) Experimental and calculated XRD spectra of (TeFBTT)_2_PbI_4_.


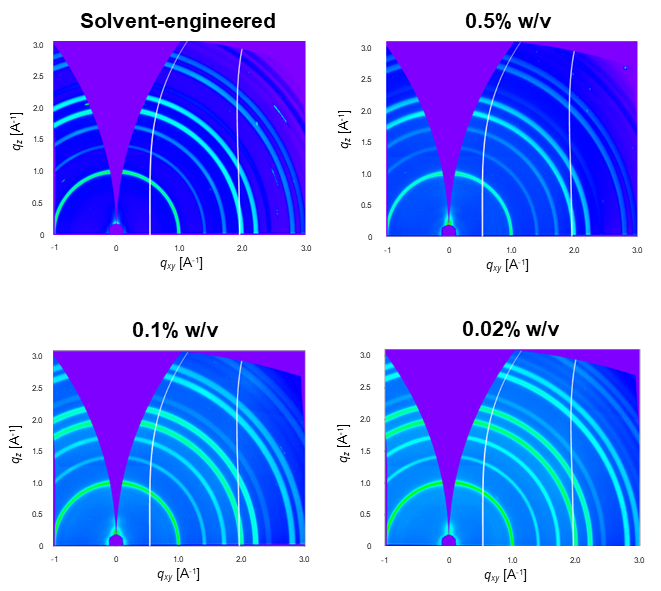


Supplementary Fig. 6. GIWAXS analysis. GIWAXS patterns of perovskite films treated with different TeFBTT concentrations.

**
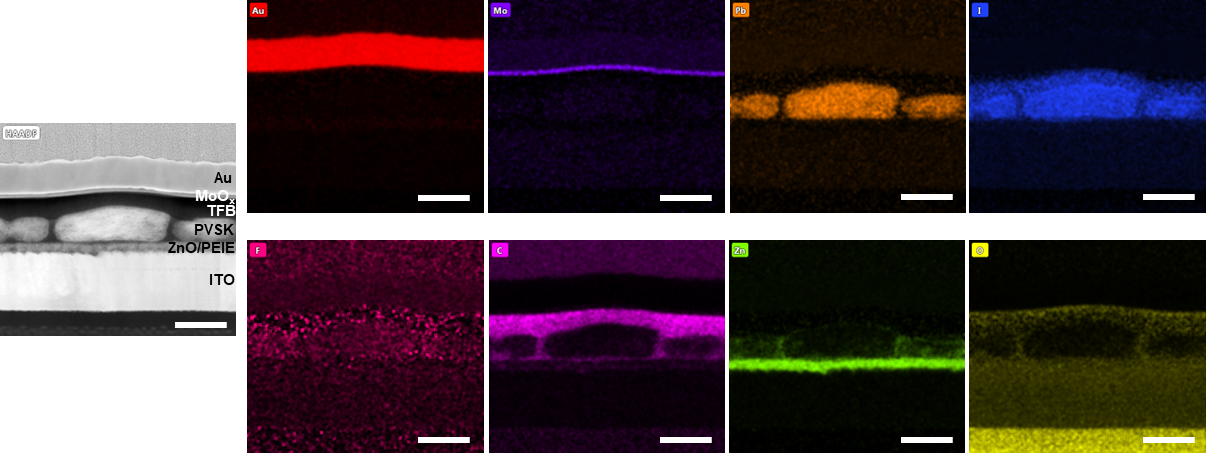
**

**Supplementary Fig. 7. TEM analysis (TeFBTT-treated device).** Cross-sectional TEM and EDS mapping images of the TeFBTT-treated device (scale bar: 100 nm).

**Note:** Since the 2D layer is very thin, it is challenging to observe it from the cross-sectional TEM image. However, the fluorine (F) signal from TeFBTT was predominantly observed around the surface of perovskite grains in the EDS mapping.

**
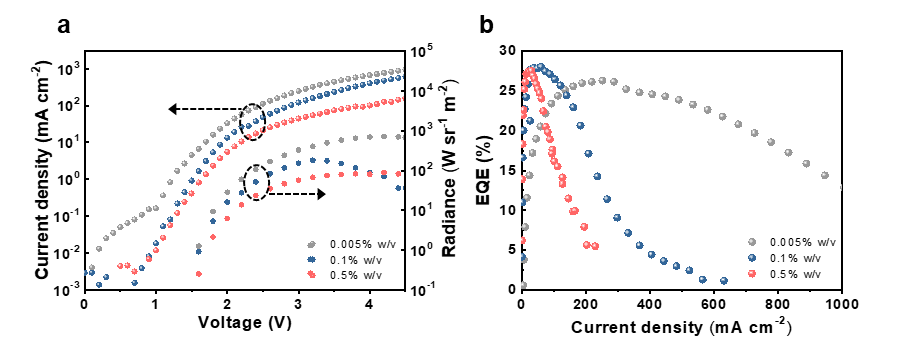
**

**Supplementary Fig. 8. Device characteristics of PeLEDs with TeFBTT.** (a) *J*-*V*-*R* curves and (b) EQE characteristics of PeLEDs with varying TeFBTT concentrations.


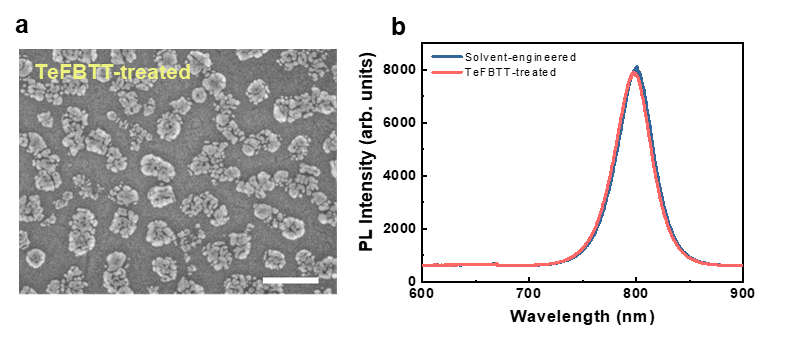


**Supplementary Fig. 9. Characteristics of TeFBTT-treated perovskite film** (a) SEM image of the perovskite film with the optimized TeFBTT concentration (0.02% w/v) (scale bar: 2 μm). (b) PL spectra of perovskite films with solvent-engineered and TeFBTT-treated conditions.


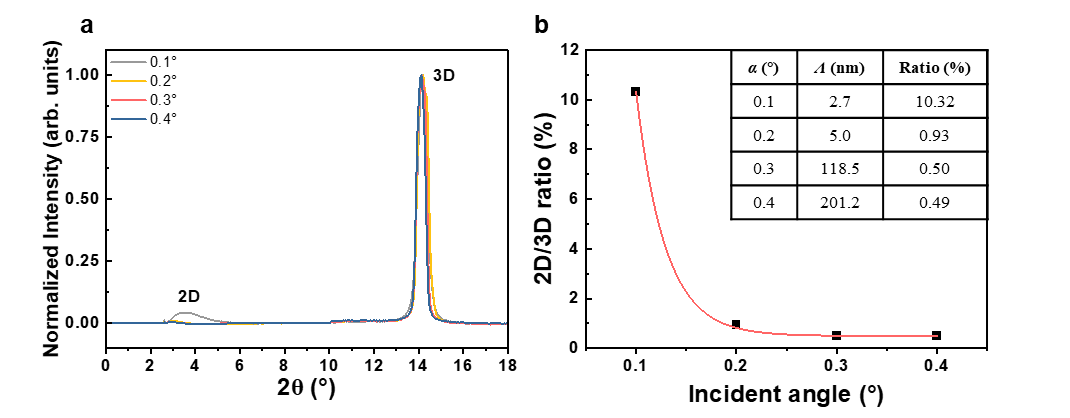


Supplementary Fig. 10. Perovskite 2D/3D phase ratio estimation. (a) Normalized 1D GIWAX patterns along qz direction. (b) Estimated 2D/3D ratio depending on X-ray incident angle (inset: X-ray penetration depth according to X-ray incident angle).

**Note**: The 2D phase ratio in the perovskite film was approximately estimated by comparing the peak areas of both 2D and 3D phases. By correlating the peak area with the X-ray penetration depth, it was confirmed that the 2D phase was predominantly present on the surface of the 3D phase, accounting for 10.3% in the 2.7 nm region and approximately 0.5% of the entire film region. The beam penetration depth ($\Lambda$) was calculated using the following equation^2^:

$$\Lambda=\frac{\lambda}{4\pi}\sqrt{\frac{2}{\sqrt{{(\alpha^{2}-\alpha_{c}^{2})}^{2}+4\beta^{2}}-(\alpha^{2}-\alpha_{c}^{2})}}$$

where $\lambda$ is X-ray wavelength (0.124 nm), $\alpha$ is X-ray incident angle, $\alpha_{c}$ is X-ray critical angle (0.23°), and *β* is X-ray absorption factor (assumed to be the same as MAPbI_3_, 2.3$\times$10 ^‑7^).


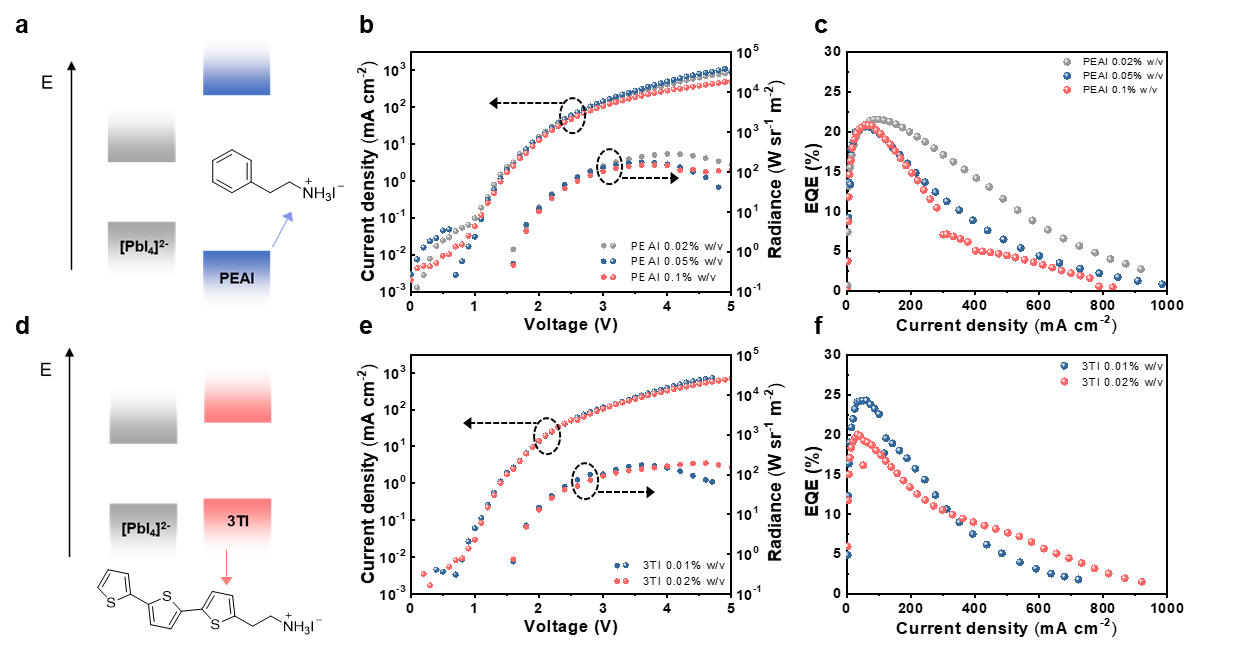


Supplementary Fig. 11. PeLEDs with other organic cations capable of forming layered 2D perovskites. (a) Chemical structure of phenylethylammonium iodide (PEAI) and energy band diagram of [PbI_4_]^2-^ and PEAI^3^. (b) *J*-*V*-*R* curves and (c) EQE characteristics of PeLEDs with PEAI. (d) Chemical structure of trithiophenylethylammonium iodide (3TI) and energy band diagram of [PbI_4_]^2-^ and 3TI^4^. (e) *J*-*V*-*R* curves and (f) EQE characteristics of PeLEDs with 3TI.

**Note**: Although PEAI forms a Type-I band alignment with inorganic motifs of 2D perovskite, it has a high energy barrier for charge-carrier injection^3,5^. 3TI forms a Type-II band alignment with inorganic motifs of 2D perovskite, which is unfavorable for PeLEDs^4,5^.


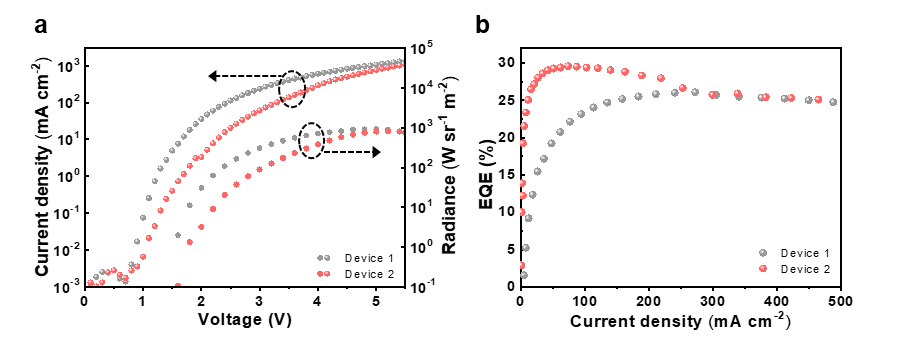


Supplementary Fig. 12. Other representative PeLEDs with TeFBTT. (a) *J*-*V*-*R* curves and (b) EQE characteristics.


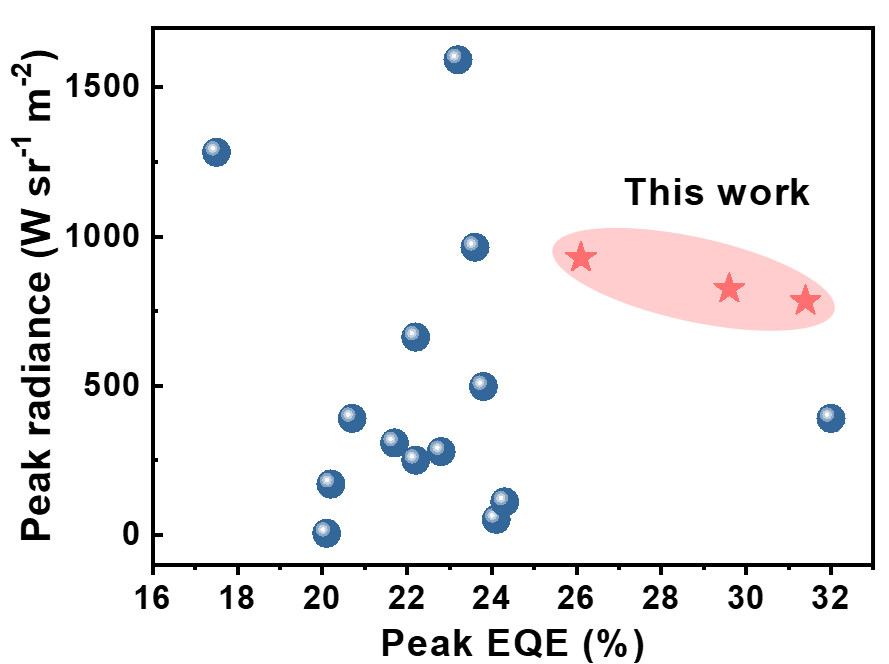


**Supplementary Fig. 13. Reported peak EQE and radiance of NIR PeLEDs** based on the data presented in Supplementary Table 1, in conjunction with this work.


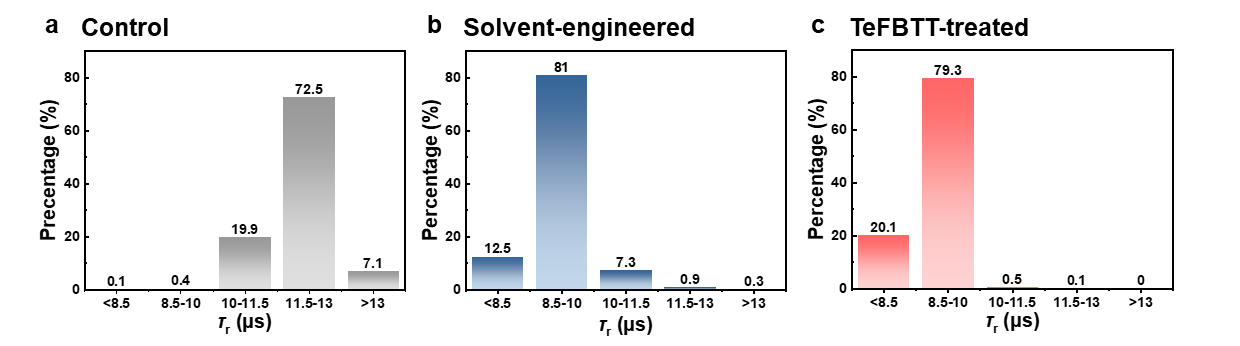


Supplementary Fig. 14. Histograms of nanoscale charge-carrier recombination lifetime mapping for perovskite films. (a) Control, (b) solvent-engineered, and (c) TeFBTT-treated films.

**
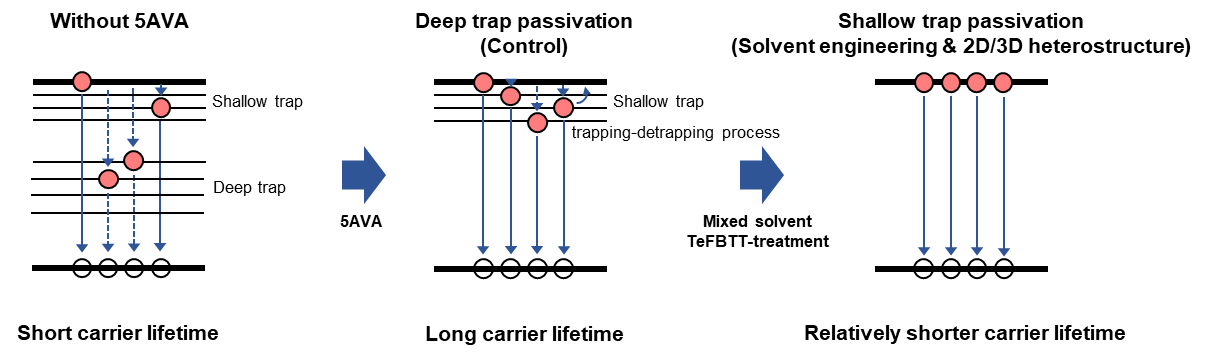
**

Supplementary Fig. 15. Defect passivation model. Schematic of the defect passivation model and carrier recombination lifetime.

**Note**: It is crucial to note that our control sample was prepared using the 5AVA additive, which implies that a significant number of defects, particularly deep-level defects, were already passivated, resulting in a moderate PLQY of approximately 70%. As the film undergoes the solvent engineering and TeFBTT treatment, shallow defects are gradually passivated (increased PLQY), which suppresses the carrier trapping-detrapping process, thereby reducing the exciton lifetime^6–10^. Additionally, solvent engineering transforms the film morphology from a continuous to discontinuous island-type structure. This morphological change introduces inter-island barriers, which could further reduce the carrier diffusion and, thereby, lifetime as well^11^.


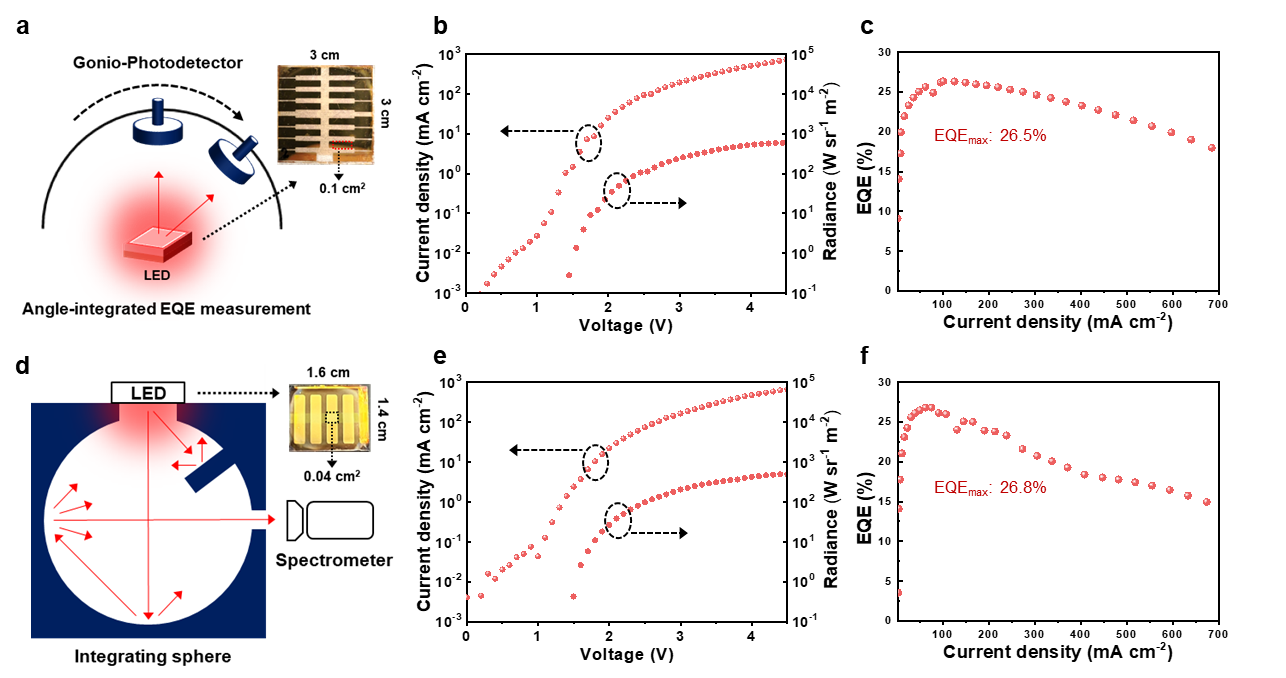


**Supplementary Fig. 16. Cross-Validation of PeLEDs.** (a) Schematic representation for angle-integrated EQE measurement in a cross-validation institution, along with a camera image of the tested device. (b) *J*-*V*-*R* curves and (c) EQE characteristics of the device for the cross-validation. (d) Schematic representation of the integrating sphere in our measurement system, accompanied by a camera image of the tested device. (e) *J*-*V*-*R* curves and (f) EQE characteristics of the device.

**
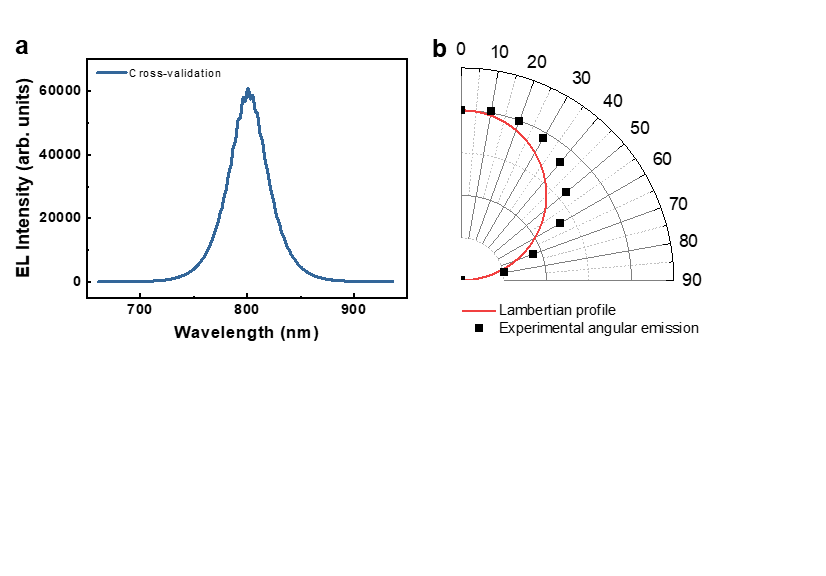
**

**Supplementary Fig. 17. Device characteristics of the cross-validated PeLED.** (a) EL spectrum and (b) ideal Lambertian profile with experimental angular emission data of the cross-validated PeLED.

**Note**: The broader emission profile observed in the experimental angular emission, compared to ideal Lambertian profile, is presumably due to the enhanced light outcoupling effect by optimized discrete island – convex dome structure.

Supplementary Table 1. Performance summary of reported NIR PeLEDs.

| EL peak (nm) | Peak EQE (%) | Peak radiance  (W sr^-1^ m^-2^) | Device structure | Year | Reference number |
| --- | --- | --- | --- | --- | --- |
| 799 | **31.4** | **784** | ITO/ZnO/PEIE/FAPbI_3_-5AVA-TeFBTT/TFB/MoO*_x_*/Au | **2024** | **This work** |
|  | **29.6** | **825** |  |  |  |
|  | **26.1** | **929** |  |  |  |
| 805 | 32.0 | 390 | ITO/ZnO/PEIE/FAPbI_3_-5AVA-PyNI- /TFB/MoO*_x_*/Au | 2024 | 12 |
| 802 | 23.6 | 964 | ITO/ZnO/PEIE/FAPbI_3_-5AVAI/TFB/MoO*_x_*/Au | 2024 | 13 |
| 789 | 23.2 | 1593 | ITO/ZnO/PEIE/FA_0.83_Cs_0.17_PbI_3_-5AVAI-Pb(SCN)_2_-AAs/ TFB/MoO*_x_*/Au | 2024 | 14 |
| 793 | 24.3 | 110.1 | ITO/ZnO/PEIE/FAPbI_3_-AEBS/ TFB/MoO*_x_*/Au | 2024 | 15 |
| 800 | 23.8 | 497 | ITO/ZnO/PEIE/FAPbI_3_-MSPE/  poly-TPD/MoO*_x_*/Au | 2023 | 16 |
|  | 22.2 | 663 |  |  |  |
| 804 | 24.1 | 51.8 | ITO/ZnO/PEIE/CdAc_2_-FAPbI_3_-5AVA/TFB/MoO*_x_*/Au | 2022 | 17 |
| 800 | 22.8 | 278.9 | ITO/ZnO/PEIE/FAPbI_3_-SFB10/  TFB/MoO*_x_*/Au | 2022 | 18 |
| 800 | 22.2 | ~250 | ITO/ZnO/PEIE/FAPbI_3_-AEAA/  TFB/MoO*_x_*/Au | 2021 | 19 |
| 789 | 17.5 | 1282.8 | ITO/ZnO/PEIE/  PPAI-FA_0.83_Cs_0.17_PbI_3_/TFB/MoO*_x_*/Au | 2021 | 20 |
| 799 | 20.2 | 170 | ITO/ZnO/PEIE/FAPbI_3_-5AVA/  poly-TPD/MoO*_x_*/Au | 2020 | 21 |
| 800 | 21.7 | 308 | ITO/ZnO/PEIE/FAPbI_3_-ODEA/TFB/MoO*_x_*/Au | 2019 | 22 |
| 803 | 20.7 | 390 | ITO/ZnO/PEIE/FAPbI_3_-5AVA/  TFB/MoO*_x_*/Au | 2018 | 23 |
| 795 | 20.1 | ~5 | ITO/MZO/PEIE/(NMA)_2_(FA)Pb_2_I_7_:poly-HEMA/TFB:PFO/MoO*_x_*/Au | 2018 | 24 |

Supplementary Note 1. Solution chemistry between 5AVA and FAI

The procedure to synthesize polycrystalline perovskite films of 5AVA-modified FAPbI_3_ was adopted from the literature^23^. However, the solution chemistry involving 5AVA and FAI has not been elucidated before. Recent results in the field of FAPbI_3_-based solar cells suggest a plausible reaction between primary amine and FAI to produce gaseous ammonium both in solution and solid state^25,26^. Inspired by these studies, and since the amino acid 5AVA is involved in our precursor solution, a detailed analysis was conducted to unveil the solution chemistry between 5AVA and FAI. In a nutshell, results in this section indicated that 1) 5AVA reacts with itself to form 2‑piperidone in DMF or DMSO which protects itself from further reaction with FAI. However, 2) formation of lactam competes with the as-predicted reaction between the primary amine from 5AVA and FAI.


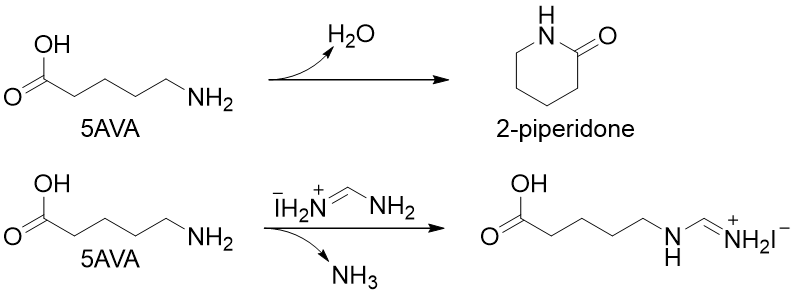


Supplementary Fig. 18. Plausible chemistry involving 5AVA. Ring-closure lactam formation vs. reaction between 5AVA and FAI to form gaseous ammonia.

**1) Ring-closure lactam formation**

First, gas chromatography mass spectra (GC-MS) were obtained from the DMF solution of 5AVA+5 equiv. in excess FAI mixture and compared with those from the DMF solution of each individual component. Note, 5AVA itself has poor solubility in DMF without the presence of excess FAI. Thus, each solution/suspension studied was heated at 60 ^o^C for 8 hours and filtered through a 0.22 µm syringe filter before GC-MS was conducted. The preparation of solutions was performed in a N_2_-filled glovebox to mimic the device fabrication process and prevent the impact from atmospheric O_2_ and moisture. The initial concentration of 5AVA was kept at 0.1 M while the final concentration after filtration was unclear, except in the 5AVA-FAI mixture case where a clear solution could be obtained. Thus, the absolute peak intensity from GC was not quantitatively analyzed. This excessive heating condition was found to induce complete transition from 5AVA to 2-piperidone, as elaborated later.

Supplementary Fig. 19. GC-MS spectra. Formation of 2-piperidone from 5AVA probed with GC-MS.

As exhibited in Supplementary Fig. 19, 2-piperidone was unambiguously identified from 5AVA/DMF solution. The presence of excess FAI in the solution were not able to consume 2‑piperidone, indicating that the formation of lactam prevents any further reaction with FAI. However, the pristine 5AVA does not exist neither in the DMF solution of 5AVA itself nor with the presence of extra FAI.

Additionally, the ^1^H and ^13^C NMR spectra of 5AVA in DMSO-d_6_ were obtained (Supplementary Fig. 20). Note, 5AVA has higher solubility in DMSO than in DMF and a clear concentrated (ca. 0.1 M) solution of 5AVA in DMSO-d_6_ could be obtained with mild heating, and thus, we were able to obtain the spectra before the complete consuption of 5AVA to form 2‑piperidone. The ^1^H signal from amide was clearly identified and correlated with other aliphatic ^1^H signals from 2-piperidone. Aliphatic ^1^H peaks from the leftover unreacted 5AVA were also identified. Furthermore, ^13^C NMR of a completely reacted solution indicated only the presence of 2-piperidone.


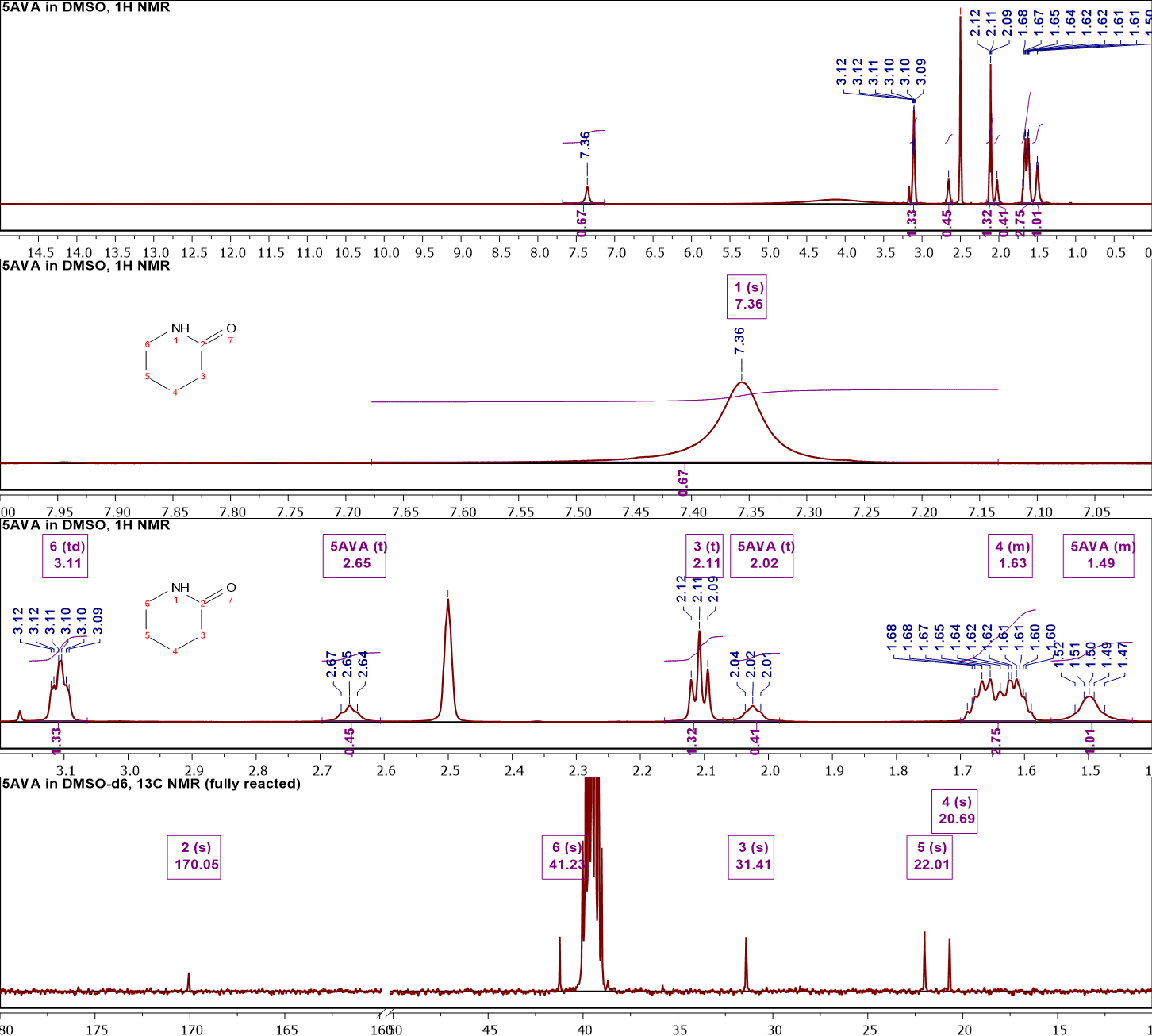


Supplementary Fig. 20. NMR spectra of 5AVA in DMSO. ^1^H spectra were obtained before the complete reaction, while the ^13^C spectrum was obtained after the complete reaction.

In summary, the combined results from GC-MS and NMR indicated that the self-reaction of 5AVA to form 2-piperidone takes place in DMF or DMSO, driven by mild heating at 60 ^o^C. 2‑piperidone is inert to further reaction with FAI.

**2) Formation of gaseous ammonia**

To probe the formation of gaseous ammonia, 5AVA (14.3 mg) and FAI (140 mg) were dissolved in DMSO (1 mL). Upon mild heating at 60 ^o^C, the production of basic gas could be probed by a combination of a pH strip test and an HCl fuming test. A similar phenomenon could be observed when the solution was prepared in a N_2_-filled glovebox whether DMF or DMSO were used as solvents.


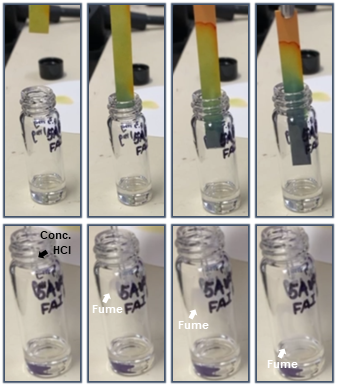


Supplementary Fig. 21. Reaction between FAI and 5AVA in DMSO probed with (top) pH strips and (bottom) production of white fume with HCl vapor.

As 2-piperidone cannot react with FAI, the formation of gaseous ammonia is likely a result of the reaction between FAI and the primary amine in leftover 5AVA, as similarly reported previously^25,26^. Unfortunately, the targeted product predicted from Supplementary Fig. 18 could not be identified from GC-MS.

Supplementary Information for organic synthesis

Supplementary Fig. 22. Synthetic scheme for TeFBTT.

**NMR spectra**

**2-bromo-3-ethylthiophene**

*^1^H NMR (400 MHz, CDCl_3_)*

*δ 7.19 (d, J = 5.6 Hz, 1H), 6.82 (d, J = 5.6 Hz, 1H),*

*2.59 (q, J = 7.6 Hz, 2H), 1.19 (t, J = 7.6 Hz, 3H).*

**(BrFBTT-Boc)**

*tert*-butyl (2-(5-(7-bromo-5,6-difluorobenzo[*c*][1,2,5]thiadiazol-4-yl)thiophen-2-yl)ethyl)carbamate

*^1^H NMR (400 MHz, CDCl_3_)*

*δ 8.11 (d, J = 3.8 Hz, 1H), 7.00 (d, J = 3.9 Hz, 1H),*

*3.48 (d, J = 4.4 Hz, 2H), 3.12 (t, J = 6.7 Hz, 2H), 1.45 (s, 9H).*

**(TeFBTT-Boc)**

*tert*-butyl (2-(5-(7-(3-ethylthiophen-2-yl)-5,6-difluorobenzo[*c*][1,2,5]thiadiazol-4-yl)thiophen-2-yl)ethyl)carbamate

*^1^H NMR (400 MHz, CDCl_3_)*

*δ 8.16 (d, J = 3.1 Hz, 1H), 7.55 (d, J = 5.1 Hz, 1H), 7.15 (d, J = 5.3 Hz, 1H),*

*7.02 (d, J = 3.3 Hz, 1H), 4.74 (s, 1H), 3.53 – 3.46 (m, 2H), 3.13 (t, J = 6.7 Hz, 2H),*

*2.54 (q, J = 6.8 Hz, 2H), 1.46 (s, 9H), 1.20 (t, J = 7.7 Hz, 3H).*

**(TeFBTT, or TeFBTT-NH_3_I)** 2-(5-(7-(3-ethylthiophen-2-yl)-5,6-difluorobenzo[*c*][1,2,5]thiadiazol-4-yl)thiophen-2-yl)ethan-1-aminium iodide

*^1^H NMR (500 MHz, DMSO-d_6_)*

*δ 8.15 (d, J = 3.8 Hz, 1H), 7.82 (d, J = 5.2 Hz, 1H), 7.79 (s, 3H),*

*7.23 (d, J = 5.2 Hz, 2H), 3.21 (q, J = 3.9 Hz, 4H), 2.48 (t, 2H), 1.13 (t, J = 7.6 Hz, 3H).*

*^19^F NMR (470 MHz, DMSO-d_6_, uncalibrated)*

*δ -128.41 (d, J = 19.9 Hz), -129.78 (d, J = 19.1 Hz).*

*^13^C NMR (126 MHz, DMSO-d_6_) δ 150.47 (d, J = 7.7 Hz), 150.47 (dd, J = 209.7, 19.2 Hz), 148.44 (dd, J = 214.2, 19.2 Hz), 147.89 (d, J = 9.0 Hz), 145.00, 143.51 (d, J = 6.2 Hz), 131.59 (d, J = 7.9 Hz), 129.26, 128.47, 128.42, 126.86, 122.68, 112.50 (d, J = 12.4 Hz), 111.02 (d, J = 16.4 Hz), 27.43 (s, 2C), 22.15, 14.59.*


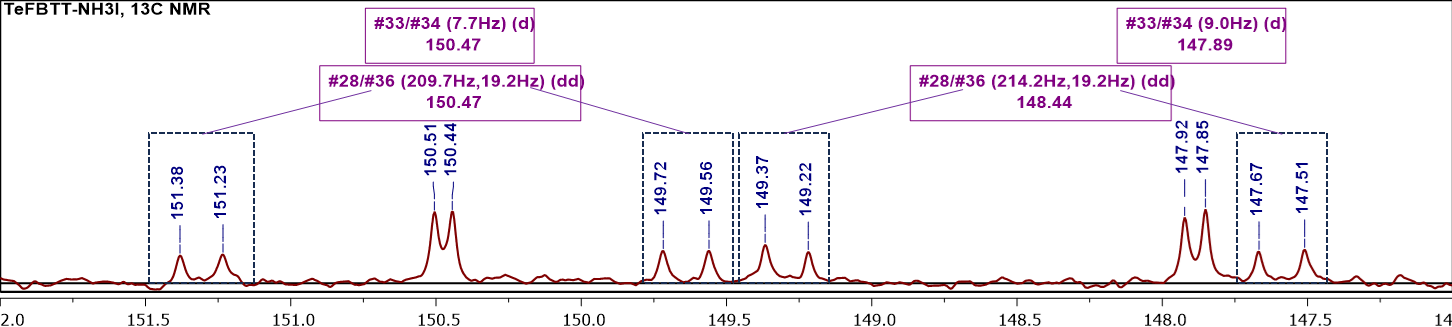


Supplementary Table 2. Crystal data and structure refinement for (TeFBTT)_2_PbI_4_.

| CCDC# | 2305444 |
| --- | --- |
| Moiety formula | (C_18_H_16_F_2_N_3_S_3_)_2_PbI_4_ |
| Empirical formula | C_36_H_32_F_4_I_4_N_6_PbS_6_ |
| Formula weight | 1531.82 |
| Temperature [K] | 150(2) |
| Crystal system | monoclinic |
| Space group (number) | $P2_{1}/c$ (14) |
| *a* [Å] | 30.918(11) |
| *b* [Å] | 8.539(2) |
| *c* [Å] | 8.892(2) |
| α [°] | 90 |
| β [°] | 95.659(19) |
| γ [°] | 90 |
| Volume [Å^3^] | 2336.1(11) |
| Z | 2 |
| *ρ*_calc_ [gcm^-3^] | 2.178 |
| *μ* [mm^-1^] | 30.710 |
| *F*(000) | 1432 |
| Crystal size [mm^3^] | 0.005×0.100×0.120 |
| Crystal colour | orange |
| Crystal shape | plate |
| Radiation | Cu*K_α_* (λ=1.54178 Å) |
| 2θ range [°] | 5.74 to 140.07 (0.82 Å) |
| Index ranges | -38 ≤ h ≤ 37 -10 ≤ k ≤ 10 -10 ≤ l ≤ 10 |
| Reflections collected | 16238 |
| Independent reflections | 16238  *R*_int_ = 0.0920 *R*_sigma_ = 0.1444 |
| Completeness to θ = 67.679° | 99.2% |
| Data / Restraints / Parameters | 16238 / 979 / 474 |
| Goodness-of-fit on *F*^2^ | 1.045 |
| Final *R* indexes [*I*≥2σ(*I*)] | *R*_1_ = 0.0952 w*R*_2_ = 0.2377 |
| Final *R* indexes [all data] | *R*_1_ = 0.1511 w*R*_2_ = 0.2830 |
| Largest peak/hole [eÅ^-3^] | 3.12/-2.98 |

Refinement details

The crystal under investigation was found to be non-merohedrally twinned. The orientation matrices for the two components were identified using the program Cell_Now^27^, with the two components being related by a 180^o^ rotation around the reciprocal a-axis. The transformation matrix found was:

[1.000 0.000 0.685]

[0.000 -1.000 0.000]

[0.000 0.000 -1.000]

Integration proved problematic due to excessive overlapping of reflections, leading to a large number of rejected reflections. Attempts were made to adjust integration parameters to avoid excessive rejections (through adjustments to integration queue size, integration box slicing and twin overlap parameters, and omission of box size optimization), which led to less but still substantial numbers of rejected reflections.

With no complete data set obtainable through simultaneous integration of both twin domains, the data were instead handled as if not twinned, with only the major domain integrated, and converted into an hklf 5 type format hkl file after integration using the "Make HKLF5 File" routine as implemented in WinGX^28^. The twin law matrix was used as obtained from SAINT, see above^29^. The Overlap R1 and R2 values used were 0.45, i.e., reflections with a discriminator function less or equal to overlap radius of 0.45 were counted overlapped, all others as single. The discriminator function used was the "delta function on index non-integrality". No reflections were omitted.

The structure was solved using direct methods with the hklf 4 type file and was refined using the hklf 5 type file, resulting in a BASF value of 0.261(5).

No Rint value is obtainable for the hklf 5 type file using the WinGX routine^28^. The value from the dataset prior to correction for twinning is given instead^30^.

The organic cation was found to be disordered by a rotation of the central PhN_2_S ring, with the disorder extending to all other cation atoms other than the ammonium N atom. The two disordered moieties were restrained to have similar geometries, and chemically equivalent bonds were restrained to be similar. sp^2^ hybridized segments of the cation were restrained to be close to planar. Uij components of ADPs for disordered atoms closer to each other than 2.0 Å were restrained to be similar. Subject to these conditions, the occupancy ratio refined to 0.485(19)-0.515(19).

No indication was found for presence of a larger unit cell ordered phase, or a lower symmetry ordered phase.

Supplementary Note 2. Grain analysis from SEM and AFM

**1. Determining the average height (*H*) of perovskite layers from AFM topography**

Related to Fig. 1a and Supplementary Fig. 1.

Accurate determination of *H* requires subtracting the average height of the underlying layer as the baseline from the average height of an AFM topography image. The discontinuous film morphology enables the extraction of this baseline by averaging the height from the “black voids”. As illustrated below (Supplementary Fig. 23) with a representative topography from DMF:NMP = 10:1, selecting the topography below ca. 56 nm allows the determination of baseline height.


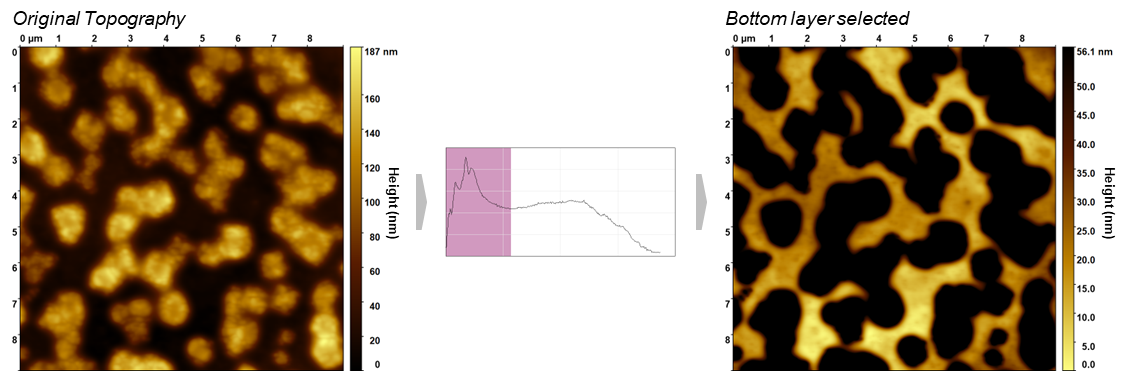


Supplementary Fig. 23. The average height (*H*) determination. A representative AFM topography image from the film with DMF:NMP = 10:1 condition.

**2. Determining the convex height (*h*_s_) atop the TFB layer**

Related to Supplementary Fig. 4 and used to determine the experimental *h*_s_ for optical simulation in Fig. 2a.

AFM topography of the perovskite/TFB double layer is acquired, followed by extracting 10 line profiles from each topography. Each line profile contains the height variation on top of TFB layer created by the discontinuous film morphology of the underlying perovskite polycrystalline film.


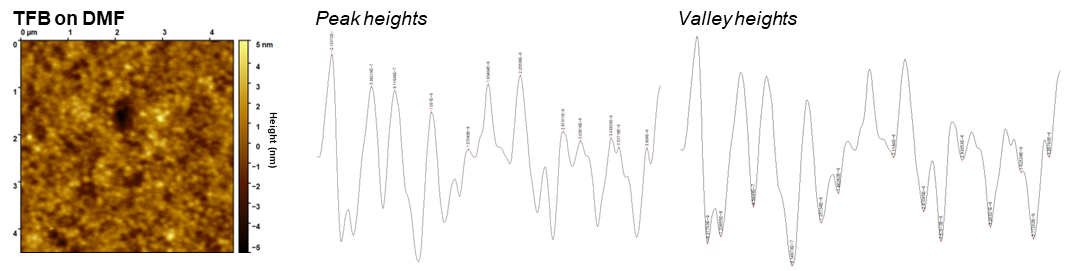


Supplementary Fig. 24. The convex height (*h*_s_) determination. A representative line profile extracted from a topography image of perovskite/TFB double layer.

The image above (Supplementary Fig. 24) illustrates a representative line profile extracted from a topography image of TFB on perovskite with the DMF condition (control). Height values of every peak and valley were obtained using OriginLab software. The average *h*_s_ is thus obtained by subtracting the mean valley height from the mean peak height.

**3. Determining average grain size and packing density (*α*) from SEM topography**

Packing density of each condition is included in Fig. 1a; average grain size is used to construct the optical simulation model in Fig. 2a.

Particle analysis was conducted in ImageJ software using the topography images obtained from SEM. The entire process is illustrated below (Supplementary Fig. 25) with the control film (DMF), which exhibited much higher *α* compared with solvent-engineered films (Fig. 1a) and made it most difficult to separate grains from each other. First, the original image was denoised with a fast Fourier transform (FFT) bandpass filter to select structures >5 pixels by removing noise signals at the high frequency regime. Thresholding was then applied to the denoised image to differentiate grains from background. The binary image produced was applied with a watershed function to automatically separate close grains that were not able to be separated by the threshold. Finally, the built-in analyze particles function was used to extract the average particle size and *α*.


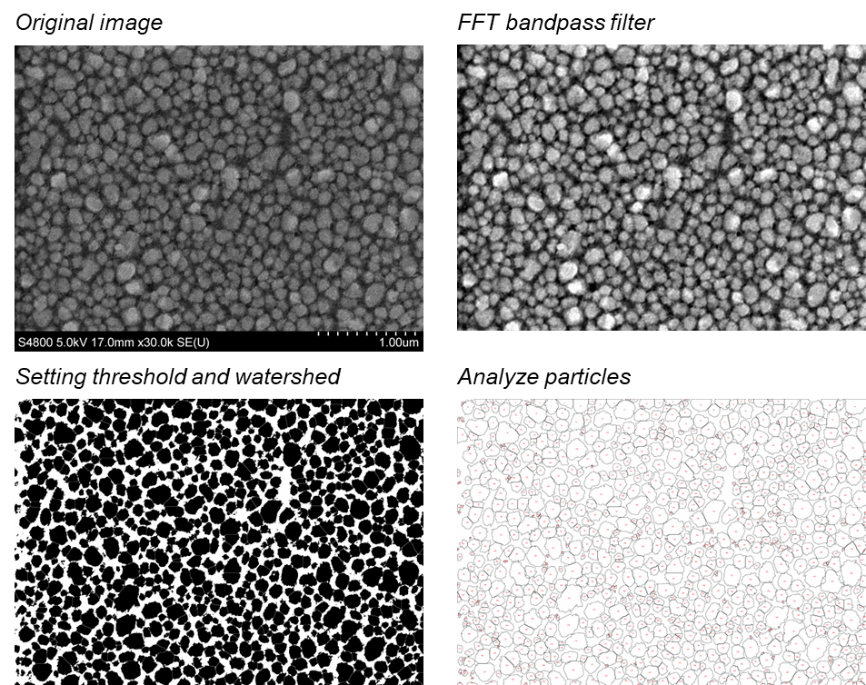


Supplementary Fig. 25. Particle analysis process. SEM image processing for grain size and distribution analysis.

Supplementary Note 3. Power density calculation for PLQY measurements

The spot size was measured by shooting laser at the sample location on a measurement card with scales. The spatial distribution of laser spot intensity was then extracted in the horizontal and vertical directions. The spatial distribution was assumed to be gaussian with a little deviation. Thus, the power density was calculated as $\frac{2P_{0}}{A_{1/e^{2}}}$, where $P_{0}$ was the total power measured by a power meter, and $A_{1/e^{2}}$ was the area estimated with an eclipse function using the horizontal and vertical diameter at the $P_{0}/e^{2}$position.


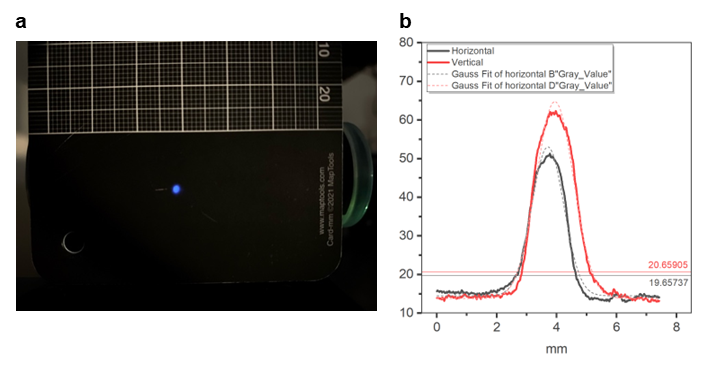


Supplementary Fig. 26. Laser power density details. (a) Laser spot camera image and (b) spatial distribution of laser spot intensity.

Supplementary Note 4. Optical simulations

**1. Model construction and general simulation considerations**

Device and perovskite (pvsk) film morphology was modeled in 3D-FDTD simulation with a method modified from the previously reported literature^23^. A single cell (length: *P*) contains one perovskite grain simplified as a tetragonal block embedded in TFB with defined length (*l*) and height (*H*). The packing density (*α*) therefore equals (*l*/*P*)^2^. These parameters were extracted from SEM and AFM as mentioned in Supplementary Note 2. The curvature atop TFB was simplified as a “convex dome” defined by the convex height (*h*_s_). The thickness of TFB (50 nm) in the model refers to the distance from the top of perovskite grains to the top of the convex dome atop TFB. Gap between the perovskite grains was assumed to be filled with TFB.

It is important to clarify that the perovskite block in the cell is the only dipole source (i.e., the emitter). Also, no periodic boundary was used in our simulation. Hence, if the simulation scale extends beyond a single cell, the periodically or randomly distributed blocks nearby are considered passive structures, while the dipole source is placed as close as possible to the center. Multiple simulations with different polarizations were carried out to simulate the incoherent isotropic light source in a 4×4 uniformly distributed dipole source in one perovskite region.

Localized refined meshes (Δ*x*, Δ*y* and Δ*z*) were applied. A mesh size of 2 nm is applied in all three directions in the dipole source region and Δ*z* = 1 nm in the convex structures. A perfect match layer boundary condition is used at the glass substrate and all *x*,*y* directions. A metal boundary condition is applied at the gold layer. Lumerical’s far-field analysis group is implemented to calculate the far-field out-coupling power using the near-field power at the glass-ITO interface. The theoretical out-coupling efficiency is then calculated with the division of out-coupling power by the source power obtained inside the dipole power box. We chose 800 nm as the wavelength of interest. Refractive index of Au, MoO*_x_*, TFB, perovskite, ZnO/PEIE, ITO, and glass were extracted from the ellipsometer measurement (Supplementary Fig. 27). Note the imaginary part of the refractive index of perovskite is ignored in the simulation.


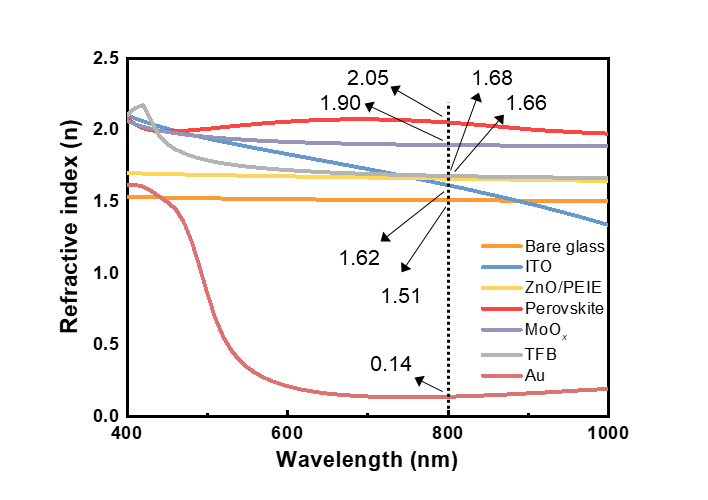


Supplementary Fig. 27. Refractive index spectra used for optical simulations. The refractive index spectra of the layers in PeLEDs were obtained through ellipsometry.

Subsequently, three types of simulations were carried out (Supplementary Fig. 28):


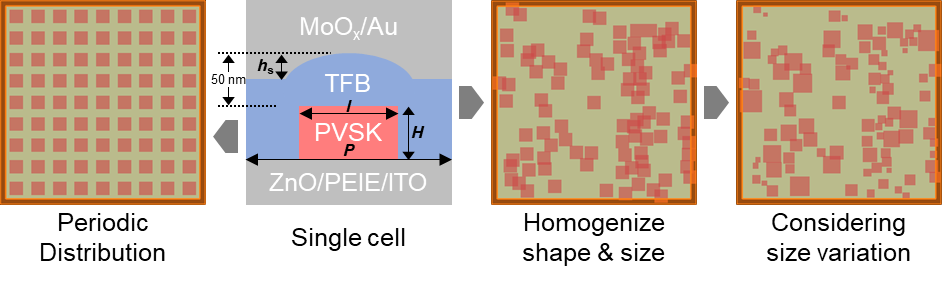


Supplementary Fig. 28. Simulation models. Schematics of three types of optical simulations.

**1. Single-cell simulation** to study the discrete island and convex dome effect

A single cell with was used to eliminate the effect of nearby grains. Thus, outcoupling efficiencies (OCE) in Fig. 2a and b do not represent those from the real device.

**2. Periodic grain distribution**

We used 9**×**9 cells for 3D-FDTD simulations, with each cell parameter set specified in Supplementary Table 3. As mentioned above, the emitting dipole was placed in the center.

Supplementary Table 3. Simulation parameters and results from the periodic model.

| *P* (nm) | *l* (nm) | *H* (nm) | *h­*_s_ (nm) | *α* | OCE (%) | Comments |
| --- | --- | --- | --- | --- | --- | --- |
| 120 | 97 | 15.5 | 1.49 | 0.66 | 26.1 | Based on DMF films |
| 259 | 185 | 69.4 | 7.52 | 0.51 | 32.8 | Based on DMF:NMP = 14:1 films |
| 500 | 200 | 86.1 | 39.6 | 0.16 | 39.1 |  |
| 500 | 312 | 86.1 | 39.6 | 0.39 | 40.4 | Based on DMF:NMP = 10:1 films |
| 500 | 355 | 86.1 | 39.6 | 0.49 | 39.9 |  |
| 500 | 407 | 86.1 | 39.6 | 0.64 | 38.9 |  |
| 500 | 450 | 86.1 | 39.6 | 0.81 | 37.7 |  |
| 500 | 475 | 86.1 | 39.6 | 0.90 | 36.7 |  |
| 500 | 312 | 40 | 39.6 | 0.39 | 37.8 |  |
| 500 | 312 | 60 | 39.6 | 0.39 | 39.8 |  |
| 500 | 312 | 100 | 39.6 | 0.39 | 40.1 |  |

**3. Random grain distribution**

Compared to the periodic grain distribution, perovskite crystals might randomly cluster together in the actual film morphology. Thus, some grains are no longer discrete, and their convex domes can be connected. To model the effect of random distribution and clustering, we selected 6 sections from the SEM image of the DMF:NMP = 10:1 film and mapped the specific distribution of grains and/or considered the various sizes of each grain. Each section was 4.5×4.5 μm^2^ large to match the size of the 9×9 supercell used in the periodic model simulation. In other words, *α*, *H*, *h*_s_ were maintained at 0.39, 86.1 nm, 39.6 nm, respectively, and each grain was still assumed to be a tetragonal block. This process was illustrated in Supplementary Fig. 29, where S1–S3 were selected where the emitter did not contact any other grains, while S4–6 were selected where the emitters formed clusters with nearby grains. Results indicated very stable OCE ~40% from all 6 structures, whether the varying size of each grain was considered. (Supplementary Table 4).


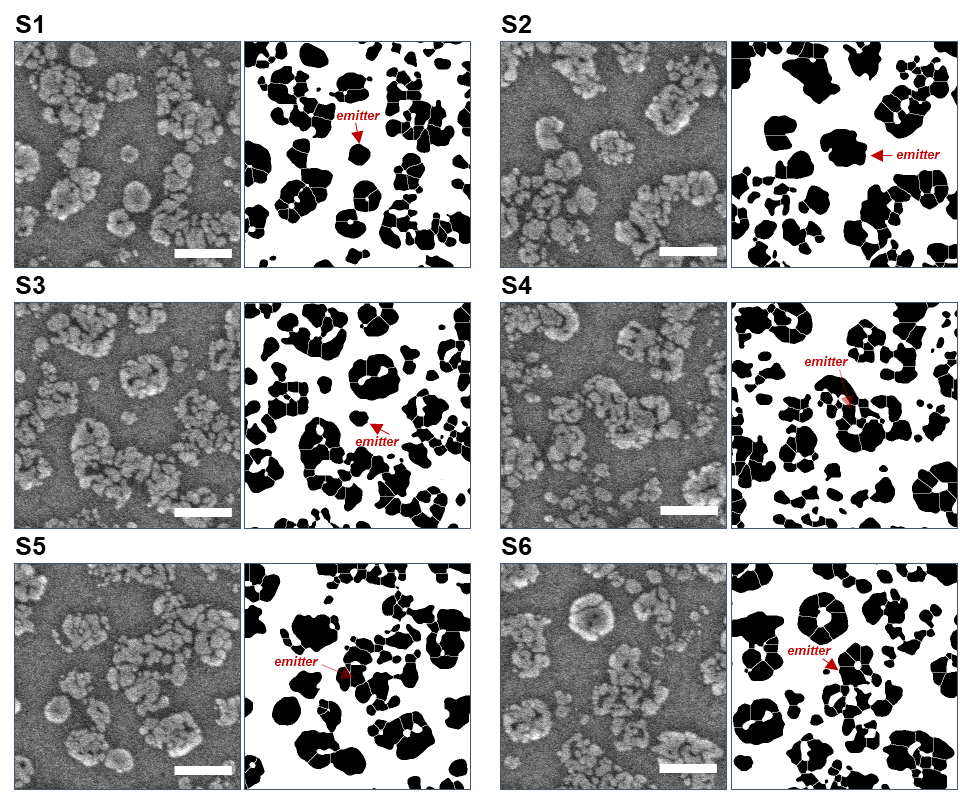


Supplementary Fig. 29. Specific SEM sections used for the randomly distributed model. Emitters at different locations were selected for comparison (scale bar: 1 μm).

Supplementary Table 4. Simulation results from the randomly distributed model.

|  | S1 | S2 | S3 | S4 | S5 | S6 |
| --- | --- | --- | --- | --- | --- | --- |
| Homogenized shape & size | 39.8% | 38.6% | 39.9% | 39.6% | 38.2% | 38.4% |
| Considering size distribution | 39.6% |  | 40.1% | 39.4% | 38.1% |  |

**Supplementary References**

1. Silver, S., Yin, J., Li, H., Brédas, J. & Kahn, A. Characterization of the Valence and Conduction Band Levels of *n* = 1 2D Perovskites: A Combined Experimental and Theoretical Investigation. *Adv. Energy Mater.* **8**, 1703468 (2018).

2. Steele, J. A. *et al.* How to GIWAXS: Grazing Incidence Wide Angle X‐Ray Scattering Applied to Metal Halide Perovskite Thin Films. *Adv. Energy Mater.* **13**, (2023).

3. Shi, E. *et al.* Two-dimensional halide perovskite lateral epitaxial heterostructures. *Nature* **580**, 614–620 (2020).

4. Park, J. Y. *et al.* Thickness control of organic semiconductor-incorporated perovskites. *Nat. Chem.* **15**, 1745–1753 (2023).

5. Wang, K. *et al.* Lead-Free Organic-Perovskite Hybrid Quantum Wells for Highly Stable Light-Emitting Diodes. *ACS Nano* **15**, 6316–6325 (2021).

6. Leijtens, T. *et al.* Carrier trapping and recombination: the role of defect physics in enhancing the open circuit voltage of metal halide perovskite solar cells. *Energy Environ. Sci.* **9**, 3472–3481 (2016).

7. Rodà, C. *et al.* Understanding Thermal and A‐Thermal Trapping Processes in Lead Halide Perovskites Towards Effective Radiation Detection Schemes. *Adv. Funct. Mater.* **31**, (2021).

8. Srivastava, S. *et al.* Advanced spectroscopic techniques for characterizing defects in perovskite solar cells. *Commun. Mater.* **4**, 52 (2023).

9. Musiienko, A. *et al.* Deciphering the effect of traps on electronic charge transport properties of methylammonium lead tribromide perovskite. *Sci. Adv.* **6**, 6393–6404 (2020).

10. Li, Y. *et al.* Shallow traps-induced ultra-long lifetime of metal halide perovskites probed with light-biased time-resolved microwave conductivity. *Appl. Phys. Rev.* **10**, (2023).

11. Chuliá-Jordán, R. & Juarez-Perez, E. J. Short Photoluminescence Lifetimes Linked to Crystallite Dimensions, Connectivity, and Perovskite Crystal Phases. *J. Phys. Chem. C* **126**, 3466–3474 (2022).

12. Li, M. *et al.* Acceleration of radiative recombination for efficient perovskite LEDs. *Nature* **630**, 631–635 (2024).

13. Li, Z. *et al.* Eliminating the Adverse Impact of Composition Modulation in Perovskite Light‐Emitting Diodes toward Ultra‐High Brightness and Stability. *Adv. Mater.* **36**, 2313981 (2024).

14. Li, Z. *et al.* Grain orientation management and recombination suppression for ultra-stable PeLEDs with record brightness. *Joule* **8**, 1176–1190 (2024).

15. Sun, X. *et al.* Regulating Surface-Passivator Binding Priority for Efficient Perovskite Light-Emitting Diodes. *Adv. Mater.* **36**, 2400347 (2024).

16. Sun, Y. *et al.* Bright and stable perovskite light-emitting diodes in the near-infrared range. *Nature* **615**, 830–835 (2023).

17. Liu, Y. *et al.* Synergistic passivation and stepped-dimensional perovskite analogs enable high-efficiency near-infrared light-emitting diodes. *Nat. Commun.* **13**, 7425 (2022).

18. Guo, B. *et al.* Ultrastable near-infrared perovskite light-emitting diodes. *Nat. Photon.* **16**, 637–643 (2022).

19. Zhu, L. *et al.* Unveiling the additive-assisted oriented growth of perovskite crystallite for high performance light-emitting diodes. *Nat. Commun.* **12**, 5081 (2021).

20. Guo, Y. *et al.* Phenylalkylammonium passivation enables perovskite light emitting diodes with record high-radiance operational lifetime: the chain length matters. *Nat. Commun.* **12**, 644 (2021).

21. Zhao, X. & Tan, Z.-K. Large-area near-infrared perovskite light-emitting diodes. *Nat. Photon.* **14**, 215–218 (2020).

22. Xu, W. *et al.* Rational molecular passivation for high-performance perovskite light-emitting diodes. *Nat. Photon.* **13**, 418–424 (2019).

23. Cao, Y. *et al.* Perovskite light-emitting diodes based on spontaneously formed submicrometre-scale structures. *Nature* **562**, 249–253 (2018).

24. Zhao, B. *et al.* High-efficiency perovskite–polymer bulk heterostructure light-emitting diodes. *Nat. Photon.* **12**, 783–789 (2018).

25. Wang, M. *et al.* Ammonium cations with high pKa in perovskite solar cells for improved high-temperature photostability. *Nat. Energy* **8**, 1229–1239 (2023).

26. Jiang, Q. *et al.* Surface reaction for efficient and stable inverted perovskite solar cells. *Nature* **611**, 278–283 (2022).

27. Sheldrick, G. M. Cell_Now. Georg-August-Universität, Göttingen, Germany (2008).

28. Farrugia, L. J. *WinGX* and *ORTEP for Windows* : an update. *J. Appl. Crystallogr.* **45**, 849–854 (2012).

29. Bruker. APEX4, SAINT and SADABS. Bruker AXS Inc., Madison, Wisconsin, USA (2022).

30. Cooper, R. I., Gould, R. O., Parsons, S. & Watkin, D. J. The derivation of non-merohedral twin laws during refinement by analysis of poorly fitting intensity data and the refinement of non-merohedrally twinned crystal structures in the program *CRYSTALS*. *J. Appl. Crystallogr.* **35**, 168–174 (2002).
